# SOLID-Similar object and lure image database

**DOI:** 10.3758/s13428-019-01211-7

**Published:** 2019-02-25

**Authors:** Darya Frank, Oliver Gray, Daniela Montaldi

**Affiliations:** Division of Neuroscience and Experimental Psychology, School of Biological Sciences, University of Manchester, Manchester, UK

## Abstract

Stimulus selection is a critical part of experimental designs in the cognitive sciences. Quantifying and controlling item similarity using a unified scale provides researchers with the tools to eliminate item-dependent effects and improve reproducibility. Here we present a novel Similar Object and Lure Image Database (SOLID) that includes 201 categories of grayscale objects, with approximately 17 exemplars per set. Unlike existing databases, SOLID offers both a large number of stimuli and a considerable range of similarity levels. A common scale of dissimilarity was obtained by using the spatial-arrangement method (Exps. 1a and 1b) as well as a pairwise rating procedure to standardize the distances (Exp. 2). These dissimilarity distances were then validated in a recognition memory task, showing better performance and decreased response times as dissimilarity increased. These methods were used to produce a large stimulus database (3,498 images) with a wide range of comparable similarities, which will be useful for improving experimental control in fields such as memory, perception, and attention. Enabling this degree of control over similarity is critical for high-level studies of memory and cognition, and combining this strength with the option to use it across many trials will allow research questions to be addressed using neuroimaging techniques.

**Electronic supplementary material:**

The online version of this article (10.3758/s13428-019-01211-7) contains supplementary material, which is available to authorized users.

Stimulus–response effects are integral to empirical experiments exploring cognitive psychology and neuroscience. The selection of stimuli is a crucial aspect of experimental design and is essential to the testing of any refined mechanistic model of cognition. In spite of this, stimulus selection and classification is often reliant on the subjective judgment of the experimenter, or their intuition. Moreover, reproducibility through high-quality experimental replication is critical to the durability, longevity, and respect of psychological science, and of science more generally (Open Science Collaboration, 2015). Consequently, an index of stimulus similarity would improve experimental precision and allow the assessment of performance across standardized task variants. Quantification of item similarity will allow tighter control over item-dependent effects and enable better reproducibility. Here we introduce a new database of over 200 objects with similar lures, which enables the selection of stimuli based on a unified dissimilarity scale.

A direct application of databases such as this one is in the field of learning and memory. Recognition memory paradigms can gain substantial benefits from the control and titration of stimulus discrimination, by manipulating target–lure similarity. For example, forced choice tasks that utilize similar lures can dissociate familiarity and recollection memory processes (Migo, Montaldi, Norman, Quamme, & Mayes, 2009). Additionally, studies exploring pattern separation and its neural bases must carefully control the degree of similarity between the target and lures in order to examine hippocampal engagement (Bakker, Kirwan, Miller, & Stark, 2008; Hunsaker & Kesner, 2013; Lacy, Yassa, Stark, Muftuler, & Stark, 2011; Liu, Gould, Coulson, Ward, & Howard, 2016). Similarly, item generalization tasks often use variants of the original studied target (such as a rotated image) to assess the degree of generalization when a task is performed under different conditions and experimental manipulations (Kahnt & Tobler, 2016; Motley & Kirwan, 2012). As a result, titrated target–lure similarity would also be particularly valuable in these studies.

Such titrations may also have utility for manipulations in the fields of visual perception, attention, and object identification experiments. For example, studies examining attention deficits often use similar foils to increase attentional demands (Rapcsak, Verfaellie, Fleet, & Heilman, 1989; Rizzo, Anderson, Dawson, Myers, & Ball, 2000). Similarly, perceptual similarity can be utilized to study the prioritization of attention and attentional control in a visual search task (Nako, Wu, & Eimer, 2014; Wu, Pruitt, Runkle, Scerif, & Aslin, 2016). Studies in these fields are often forced to rely on manipulations of verbal or abstract stimuli, which may limit investigative abilities into their research questions. Poor stimulus selection can result in the misattribution of item-dependent false-positive errors as scientifically informative effects. Participants can adopt alternative strategies without the knowledge of the experimenters, leading to erroneous assumptions about task completion. These issues may be further exacerbated in neuroimaging experiments, where the environment may augment the effect of confounding variables and induce additional interference. Hout et al. (2016) directly explored the drawbacks of stimulus selection through the subjective judgment of the researchers and demonstrated the clear advantage of using a quantitative index of stimulus similarity in visual search and eye movement research. We believe that these advantages can also greatly benefit many other aspects of cognitive neuroscience research, including the field of learning and memory.

Two multidimensional-scaling databases of dissimilarity have previously been developed for object picture sets. These databases were both established using the spatial-arrangement method (first developed by Goldstone, 1994). First, Migo, Montaldi, and Mayes (2013) produced a database of 50 object sets (~ 17 images/set) with a common scale of dissimilarity. Following the spatial-arrangement method procedure, a sample of the images was rated using pairwise comparisons. These ratings were subsequently used to standardize the level of dissimilarity across sets. Critically, this step enables two image pairs from different object sets with the same quantitative degree of dissimilarity to be interpreted as equally similar. This resource, though small, approaches the current methodological gold standard for the development of a database of dissimilarity. The dissimilarity database produced by Hout, Goldinger, and Brady (2014; based on images taken from the Massive Memory Database of Konkle, Brady, Alvarez, & Oliva, 2010) is much larger than that of Migo et al. (2013); however, the dissimilarity distances of objects within a set are substantially greater. Furthermore, the sampling-based, pairwise comparison procedure used by Migo et al. (2013) was not utilized to standardize dissimilarity distances across all the sets. Therefore, the intraset dissimilarity measure cannot be utilized for accurately matching pairwise dissimilarity across sets on a common scale. This is a serious shortcoming of the database, in that it does not provide an objective comparison, or calibration, of distances obtained from different sets of objects. Therefore, two object pairs rated with equal dissimilarity distance might in fact be quite different in the objective resolution of their similarity. Overall, these features limit the applicability of these resources in fine-grained cognitive neuroscience and neuroimaging.

The range of image pair similarities in a database is also important to its utility in cognitive neuroscience experiments. The range of within-object-set dissimilarities in previous databases has been limited. Upon visual inspection, it is clear that the images within each set of the Migo et al. (2013) database are much more similar than the images used by Hout et al. (2014). The dissimilarity between a pair of images from the Migo et al. (2013) database is comparable to viewing the same item from a different perspective. In contrast, the images used by Hout et al. (2014) generally reflect somewhat different versions of items within a set. Consequently, these two databases are very distinct and are likely to represent polar extremes of the dissimilarity continuum, with a clear lack of continuity. Production of a database with an even distribution of dissimilarities across the full continuum (within and between sets) would enable more freedom in experimental design and control, as well as the opportunity for more refined hypothesis testing.

For the Similar Objects and Lures Image Database (SOLID), we aimed to (a) develop a database large enough to be employed in neuroimaging experiments that demand many trials, (b) establish a common scale of dissimilarity both within and across image sets, and (c) ensure that the database retains utility across the dissimilarities continuum. To achieve these objectives, we first developed a new set of images to complement the images available from Migo et al. (2013). We then utilized the spatial-arrangement method to establish within-object-set dissimilarity distances for these images (Exp. 1a). We also employed the spatial-arrangement method with a subset of images (Exp. 1b) from another large database of images that does not provide data on image similarity (Konkle et al., 2010). This provided an even greater breadth of similarity and of choice of object categories and images across SOLID. In Experiment 2, we standardized the dissimilarity distances taken from Migo et al. (2013) and those established in Experiments 1a and 1b using a sample of images from each object set in a pairwise rating procedure. The scaled dissimilarity indices (DI) that were produced were then validated using a forced choice memory paradigm (Exp. 3). This ensured that image dissimilarity was accurately reflected in our DI, resulting in better memory performance and reduced response times as dissimilarity increased.

## Experiment 1a: Spatial arrangement of new object images developed for SOLID

### Method

#### Participants

Twenty participants (mean age = 28.5; 14 female, six male) performed the spatial-arrangement procedure. The sample size was informed by previous research by Migo et al. (2013) using the same procedure. Participants received £7 per hour as compensation for their time. All experimental procedures were approved by the University of Manchester Research Ethics Committee and were conducted in accordance with their guidelines and regulations. Informed consent was collected for all participants prior to data collection.

#### Materials

Freely accessible and available online resources under a Creative Common license were used to collect 231 images of everyday objects, split equally across 77 object sets (i.e., three images per set). The images were converted to grayscale and presented on a white background. These 231 images were manipulated (in terms of shape, size, shade, orientation, texture, and features) using Coral PHOTO-PAINT X5 to create another 12 images per set (total 1,155 images, 15 per set). All images are provided in the supplementary materials. Images were presented in Microsoft PowerPoint on a 21-in. computer monitor (screen resolution = 1,920 × 1,080 pixels) and arranged by participants using a mouse.

#### Procedure

Participants were presented with the 15 images in each object set on each trial and instructed to arrange the images so that the distance between any image pair reflected the dissimilarity of that pair of images (Charest, Kievit, Schmitz, Deca, & Kriegeskorte, 2014; Goldstone, 1994; Migo et al., 2013). A greater distance between images indicated greater dissimilarity, irrespective of position. Participants were encouraged to use the whole screen to arrange the images into the spatial arrangement that best represented the dissimilarity across the set. We clearly articulated that this arrangement need not apply across different categories. This process enabled the collection of dissimilarity ratings between all objects within an object set in a single trial. Each testing session consisted of 77 self-paced trials and took approximately 105 min to complete. No participants took more than 120 min to complete the entire procedure.

### Results

**Fig. 1:**
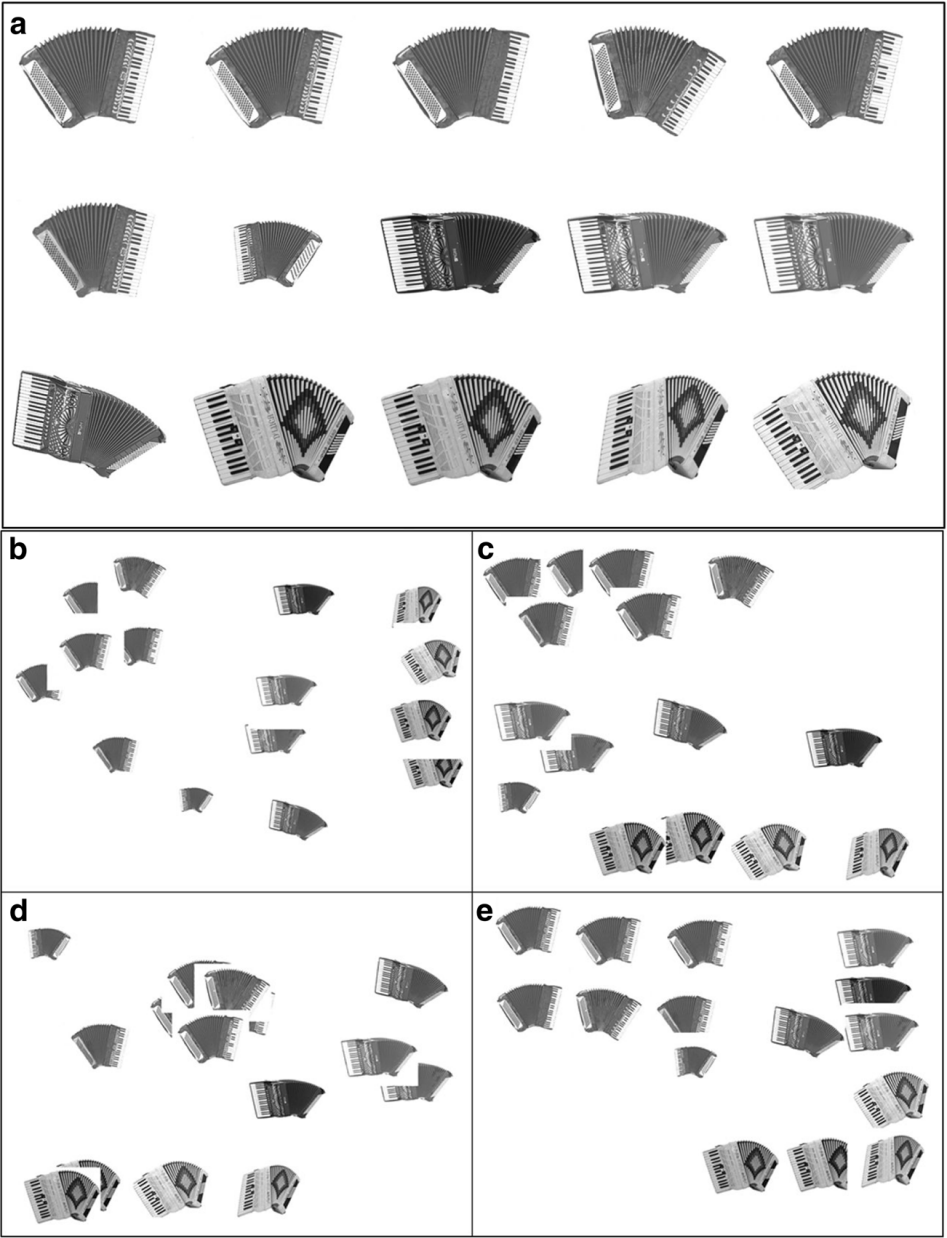
(A) The “Accordion” object set from Experiment 1a. (B) The group average 2-D multidimensional-scaling (MDS) solution. (C) One participant’s sorting map, closest to the group MDS solution. (D) Another participant’s sorting map, furthest away from the group MDS solution. (E) A participant’s sorting map midway between those in panels C and D.

Each sorting map (Fig. 1) produced an individual dissimilarity rating for every object as compared to every other object within a set. Averaging across each image-by-image comparison between participants produced a matrix of dissimilarity distances for each object set. The mean dissimilarity distance within a set ranged from between 250 (Ladybird) and 297 (Cards) pixels. As expected, there were minor differences in judgments of the dissimilarity distance of each image pair across participants. The variability of these participant-specific sorting maps produced a standard deviation for each image pair. We provide the group average dissimilarity distance for every image pairing in every set and the standard deviations of these distances in the supplementary materials.

To further examine the level of agreement between participants in sorting the objects within each set, we performed multidimensional scaling (MDS) for each set, using the PROXSCAL algorithm in SPSS (IBM, v.23). MDS is used to spatially represent the relationships between data points. Using a set number of dimensions (*k*), the goal of this analysis is to minimize the differences between the input proximities (dissimilarity distance, in this case) and the new representation of the data in *k* dimensions. We reduced our data to two dimensions by using a stress convergence value of .0001 and a maximum of 100 iterations. We then computed a dissimilarity measure, *d*, reflecting the difference between each participant’s similarity sorting and the final group average (Migo et al., 2013). Table 1 shows that the highest variation in sorting among participants was for the “Avocado” set, and the lowest was for “Balloon.”

**Table 1:** Results of the multidimensional-scaling analysis for Experiment 1a

Picture Set	*d*	*SD*	Picture Set	*d*	*SD*	Picture Set	*d*	*SD*
Accordion	0.459	0.162	Cushion	0.43	0.181	Pigeon	0.494	0.175
Acorn	0.417	0.195	Daisy	0.412	0.158	Pin	0.434	0.182
Airplane	0.433	0.2	Deer	0.47	0.157	Pipe	0.397	0.114
Artichoke	0.408	0.163	Dolphin	0.393	0.16	Rabbit	0.475	0.176
Avocado	0.593	0.212	Duck	0.442	0.186	Racket	0.486	0.137
Axe	0.465	0.172	Faucet	0.433	0.126	Radish	0.501	0.162
Balloon	0.317	0.153	Fishing rod	0.478	0.204	Real pig	0.336	0.187
Beach ball	0.572	0.175	Frog	0.44	0.128	Recliner	0.438	0.165
Bird	0.437	0.156	Garlic	0.42	0.143	Rooster	0.352	0.153
Bouquet	0.382	0.166	Ginger	0.423	0.176	Rose	0.381	0.125
Box	0.437	0.138	Giraffe	0.407	0.163	Shoe	0.463	0.167
Brush	0.408	0.155	Glasses	0.501	0.167	Shovel	0.502	0.196
Cable	0.405	0.23	Grape	0.406	0.205	Slide	0.408	0.185
Cactus	0.5	0.103	Guitar	0.432	0.169	Snail	0.398	0.125
Candle-large	0.364	0.159	Helmet	0.559	0.12	Snorkel	0.422	0.145
Car-classic	0.506	0.168	Horse	0.514	0.192	Starfish	0.534	0.14
Cards	0.518	0.128	Kangaroo	0.52	0.157	Sushi	0.472	0.146
Cat	0.518	0.216	Ladybug	0.472	0.171	Swan	0.507	0.18
Cauliflower	0.488	0.186	Luggage	0.401	0.17	Swing seat	0.546	0.181
Cedar	0.497	0.174	Mantis	0.356	0.154	Telephone	0.353	0.199
Cigarette	0.419	0.167	Meter	0.415	0.161	Train	0.412	0.159
Coke	0.429	0.181	Mug	0.445	0.184	Turtle	0.444	0.156
Compass	0.426	0.192	Panda	0.479	0.149	Tyre	0.578	0.212
Corn	0.396	0.165	Pencil	0.426	0.187	Veg	0.484	0.217
Cowboy hat	0.546	0.154	Penguin	0.424	0.184	Watermelon	0.502	0.118
Cupcake	0.473	0.168	Pepper	0.424	0.131			

## Experiment 1b: Spatial arrangement of object images from Konkle et al. (2010)

### Method

#### Participants

A new group of ten participants (mean age = 33; six male, four female) performed the spatial-arrangement procedure (original Method 1). To ensure that this sample size was sufficient, we compared the standard deviations of sorting with those from the previous experiment, as well as those from Migo et al. (2013), and found no differences between them. Participants received £7 per hour as compensation for their time. All experimental procedures were approved by the University of Manchester Research Ethics Committee and were conducted in accordance with their guidelines and regulations. Informed consent was collected for all participants prior to data collection.

#### Materials

In all, 1,275 images of objects, split equally across 75 object categories (17 images per set), were sourced from a large object image database without reference to item similarity (Brady, Konkle, Alvarez, & Oliva, 2008; Konkle et al., 2010). The images were converted to grayscale and presented on a white background.

#### Procedure

Participants were presented with the 17 images in each object set on each of the 75 trials and received the same instructions as had been given in Experiment 1a.

### Results

Each sorting map produced an individual dissimilarity rating for every object as compared to every other object within a set. Averaging across each image-by-image comparison between participants produced a matrix of dissimilarity distances for each object set. The mean dissimilarity within a set ranged from 287 (“Nunchaku”) to 328 (“Car”) pixels. As in Experiment 1, there were differences in judgments of the dissimilarity distance of each image pair across participants. The variability of these participant-specific differences produced a standard deviation for each image pair. We provide the group average dissimilarity distance for every image pairing in every set and the standard deviations of these distances in the supplementary materials.

We again performed an MDS analysis to further explore the similarity sorting techniques used across participants. The mean *d* (dissimilarity measure) and the MDS standard deviation can be found in Table 2, with “Stapler-var” showing the highest variation among participants, and “Necklace” the least.

**Table 2:** Results of the multidimensional-scaling analysis for Experiment 1b

Picture Set	*d*	*SD*	Picture Set	*d*	*SD*	Picture Set	*d*	*SD*
Backpack	0.474	0.159	Dresser	0.698	0.14	Pot	0.668	0.153
Bagel	0.444	0.115	Drum	0.561	0.143	Present	0.621	0.154
Ball-ball	0.473	0.16	Earing	0.649	0.17	Rabbit	0.695	0.164
Barbie	0.432	0.184	Extension	0.656	0.162	Razor	0.423	0.193
Beaker	0.695	0.119	Fan	0.521	0.174	Rug	0.597	0.099
Beer	0.534	0.093	Folder	0.496	0.174	Saddle	0.452	0.148
Bell	0.573	0.147	Garland	0.591	0.173	Scales	0.485	0.182
Big-car	0.544	0.232	Grater	0.521	0.18	Scissors	0.587	0.153
Binoculars	0.577	0.194	Hairband	0.526	0.164	Skates	0.495	0.211
Bonsai	0.662	0.141	Hairbrush	0.582	0.125	Sofa	0.625	0.132
Boot	0.649	0.168	Hammer	0.665	0.197	Soldier	0.662	0.156
Broom	0.596	0.133	Handfan	0.541	0.163	Speakers	0.626	0.179
Buggy	0.505	0.222	Headphones	0.546	0.182	Stapler-var	0.716	0.129
Calculator	0.647	0.106	Hourglass	0.613	0.163	Stocking	0.561	0.162
Camera	0.606	0.164	Kayak	0.588	0.215	Suit	0.402	0.174
Candle	0.559	0.175	Lamp	0.671	0.134	Swimsuit	0.572	0.187
Ceiling fan	0.666	0.142	Lipstick	0.598	0.275	Table	0.529	0.159
Chair	0.629	0.143	Mask	0.467	0.118	Telescope	0.557	0.2
Clock	0.582	0.151	Microscope	0.558	0.143	Toilet	0.465	0.179
Compass	0.564	0.149	Microwave	0.611	0.138	Torch	0.502	0.163
Doll house	0.651	0.16	MP3	0.644	0.139	Tree	0.506	0.229
Dolls	0.722	0.168	Muffin	0.583	0.163	Trousers	0.501	0.153
Donut	0.565	0.229	Necklace	0.376	0.121	Trunk	0.572	0.174
Door-key	0.552	0.176	Nunchaku	0.636	0.162	Watch	0.549	0.219
Doorknob	0.483	0.177	Padlock	0.593	0.115			

## Experiment 2: From dissimilarity distances to DI

### Method

#### Participants

A group of 23 new participants (mean age = 19.4, all female) completed the standardizing procedure in exchange for course credit. This experiment was approved by the University of Manchester Research Ethics Committee and was conducted in accordance with their guidelines and regulations. Informed consent was collected for all participants prior to data collection. The data from two participants were excluded from the analysis due to technical failures with the task.

#### Materials

The dissimilarity distance matrices resulting from the spatial-arrangement method in Experiments 1a and 1b and in Migo et al. (2013) were used to select the images for the standardization procedure. In the previous experiments, all participants had been instructed to try to use the entire screen to sort the images. As a result, the overall relative dissimilarity distances within image sets were extremely similar. However, software and hardware differences in the collection of the spatial-arrangement maps produced systematic, overall differences in the distances produced by Migo et al. (2013) and in Experiments 1a and 1b. We therefore scaled the output of the dissimilarity distances from the spatial-arrangement maps produced in Experiments 1a and 1b to match the grand average of the dissimilarity distances produced by Migo et al. (2013). This correction overcame any potential methodological differences between the experiments. These produced matched dissimilarity matrices (provided in the supplementary material) were used to select the images for Experiment 2.

**Fig. 2:**
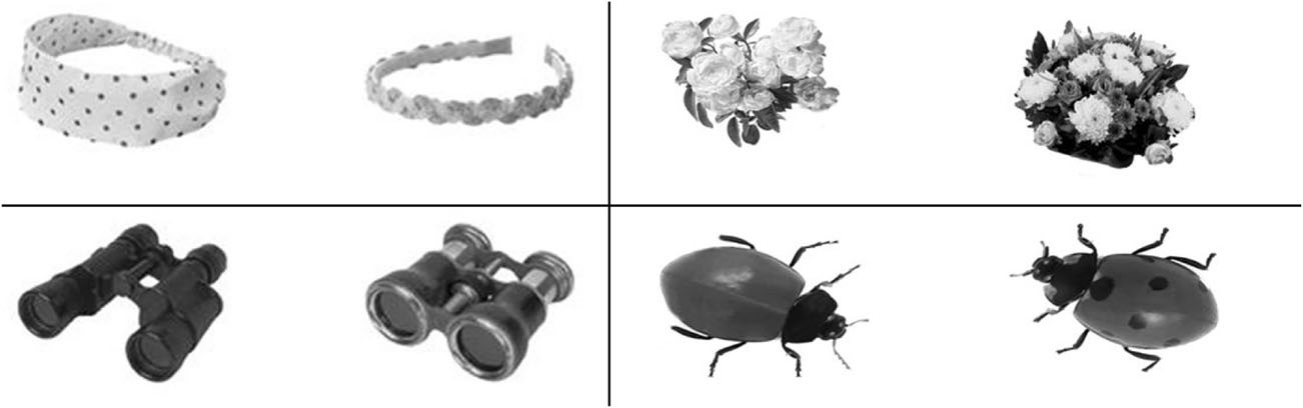
Four representative examples of image pairs used in the standardization procedure. Each pair had a very similar dissimilarity distance, resulting from Experiment 1. The rating assigned to each pair was used to moderate the similarity distances of the rest of the images in the relevant set.

We selected two images from each object set whose dissimilarity closely matched the grand average (the mean dissimilarity distance across all image comparisons) of 770 pixels (Fig. 2). Experiment 2 used 202 images pairs (one pair from every set). The mean dissimilarity distance between the selected pairs and the grand average (770) was 2.26 pixels (*SD* = 2.75).

#### Procedure

Each participant rated the similarity of each image pair on a scale from 1 to 9, where a lower number indicated that the images were more similar. The images were presented using Microsoft PowerPoint with the same materials described in Experiments 1a and 1b. Participants responded by pressing the appropriate number from 1 to 9 on the keyboard of an HP laptop. Successful standardization of the database required accurate and reliable ratings between the object categories. To prevent item-dependent effects or noise potentially induced by rating the image pairs without having seen the full range of dissimilarities, participants were required to reevaluate the first quarter of the image pair ratings. Participants were instructed that they could change any of their ratings in the reevaluation stage and that they should continue until they felt their scale of ratings was consistent across all image settings.

### Results

**Fig. 3:**
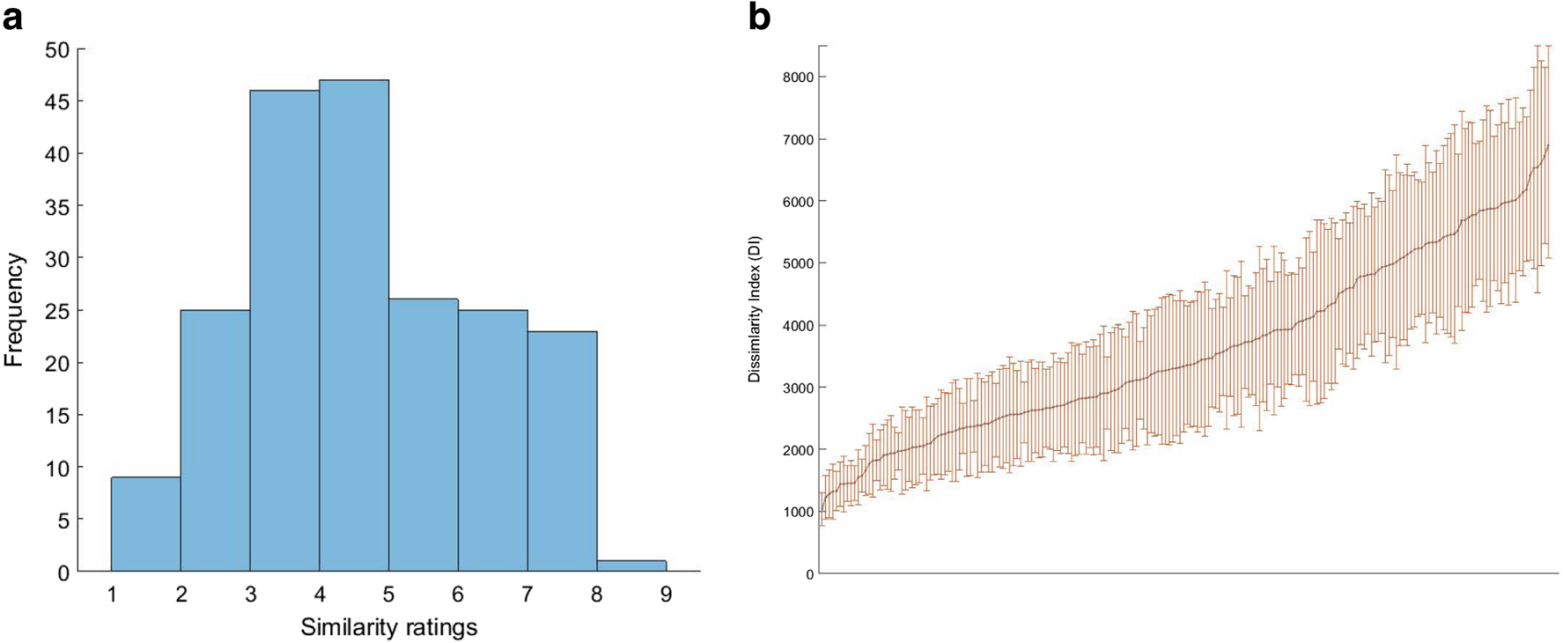
(A) The distribution of dissimilarity scores assigned to the image pairs in Experiment 2. (B) An illustration of the mean dissimilarity indexes of the object image sets.

This standardization procedure produced a dissimilarity rating for each object set. For example, the images selected within the hairband set (hairband_15 and hairband_17) received an average dissimilarity rating of 3.27. Figure 3A illustrates the distribution of average ratings across image sets. Every value in the object dissimilarity matrix was multiplied by the set’s pairwise rating. This procedure revealed the range of dissimilarities across all sets, without influencing the relative dissimilarities of all the objects within a set. Most critically, this procedure produced a set of matrices with a common scale of dissimilarity. The average dissimilarity distance across an image set was 3608.02 dissimilarity index (DI) units (*SD* = 1421.42). Figure 3B illustrates the mean DI of each image set. Importantly, an abundance of image pairs can be selected for any chosen value along the DI continuum. Furthermore, visual inspection of Fig. 3B shows that the majority of image sets show a large range of DIs across the image pairs. All tables containing the DI matrix for each object set, as well as all stimuli, are presented in the supplementary materials.

## Experiment 3: Validating SOLID in a forced choice memory paradigm

The comparability of DIs across object categories (i.e., an image pair with a DI of 700 in the “Bracelet” set is as dissimilar as an image pair with the same DI in the “Backpack” set) is crucial to the utility of SOLID. We validated the accuracy and comparability of the DIs in Experiment 3 using a forced choice memory task. The relationship between similarity and memory accuracy has been clearly described previously (Dickerson & Eichenbaum, 2010; Migo et al., 2013; Norman, 2012): As the dissimilarity between target and lure increases, an individual’s ability to identify the target over the lure improves. The selection of targets and lures based on the DIs included in SOLID should produce this pattern of behavioral performance if our data accurately scale image dissimilarity.

### Method

#### Participants

Thirty-one healthy participants completed the validation procedure. This experiment was approved by the University of Manchester Research Ethics Committee and was conducted in accordance with their guidelines and regulations. Informed consent was collected for all participants prior to data collection. Participants received course credit in exchange for their time. One participant had previously been exposed to the stimuli and was excluded from the analysis. The data from one other participant were lost due to technical difficulties during collection. All remaining participants (*N* = 29; mean age = 19.06; 27 female, two male) had normal/corrected-to-normal vision, reported no history of neurological disorder, and had not previously been exposed to the stimuli. This sample size is equivalent to that used to validate previous databases (Migo et al., 2013).

#### Materials

One image pair was selected from each object set to create target–lure pairs. The first image of the 202 image pairs was presented in the study phase and subsequently served as a target during the test phase. The second image in each object pair was used as the lure. The DI matrices developed in Experiment 2 were used to select these image pairs. Image pairs with the following target–lure DI values—1300, 2000, 2700, 3400, 4100, and 4800—were selected for use in Experiment 3. These intervals best characterized the variability in DI across the database. For counterbalancing purposes, we used three versions of the experiment, with different object sets contributing to each DI interval. This between-subjects counterbalance removed any effect of potential variability in specific item memorability.

#### Procedure

Prior to the study phase, participants were instructed to study each item carefully. We informed participants that this was a memory task and that they would be asked to distinguish between very similar images at test. The study phase consisted of 3-s presentations of single images, each presented once. Participants then engaged in mental arithmetic problems during a 5-min delay. The presentation order in both the study and test phases was random. The subsequent test phase consisted of 5-s presentations of the target and lure pair. Participants were asked to identify which of the two images they had seen previously. The experiment was presented in PsychoPy (Peirce, 2007) with the same setup as Experiments 1 and 2.

### Results

**Fig. 4:**
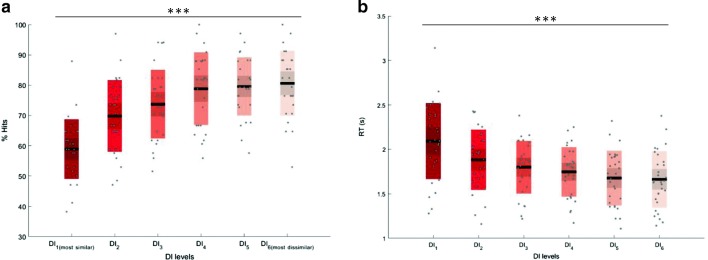
Results of the forced choice memory study. (A) This panel illustrates greater memory accuracy with decreasing levels of similarity. (B) This panel illustrates that response time (RT) results were significantly shorter with lower levels of similarity.

Experiment 3 assessed memory performance, measured by the target hit rate (calculating *d'* is not possible in forced choice paradigms, since the targets and lures are mutually dependent) over six levels of DI. A 6×3 repeated measures analysis of variance assessed differences across the six DI levels and between the three experiment versions. A significant main effect of DI level was observed [*F*(5, 130) = 37.17, *p* < .001, *η*_p_^2^ = .588]. The main effect of experiment version was not significant [*F*(2, 26) = 0.28, *p* = .755, *η*_p_^2^ = .021]. We did not observe a significant interaction between DI level and experiment version [*F*(10, 130) = 1.85, *p* = .176, *η*_p_^2^ = .125]. With increasing DI levels (target–lure dissimilarity), we observed a linear increase in memory accuracy [*F*(1, 26) = 130.74, *p* < .001, *η*_p_^2^ = .834; Fig. 4A]. Memory performance was above chance across all DI values (*p*s < .001).

Similar analyses were conducted to assess differences in response times with changes in DI levels. We observed a significant effect of DI level on response times [*F*(5, 130) = 37.51, *p* < .001, *η*_p_^2^ = .591, sphericity not assumed]. Increases in DI (target–lure dissimilarity) were accompanied by a significant linear decrease in response times [*F*(1, 26) = 72.19, *p* < .001, *η*_p_^2^ = .735; Fig. 4B].

Demonstration of the characteristic linear relationship between increasing dissimilarity and better memory performance illustrates that SOLID achieved its goal to provide a well-controlled set of object images with a wide range of similarities that can be utilized in cognitive research.

## General discussion

In the present study, we have presented a series of experiments that established a large set (3,498 images, 201 sets; mean of 17.4 [range 13–25] images per set) of everyday object images with known, quantitative similarity information. A spatial-arrangement procedure (Goldstone, 1994; Hout, Goldinger, & Ferguson, 2013) quickly and efficiently provided similarity data on all images from all participants. Image pairs with a common dissimilarity score were then selected from each object set. A pairwise similarity rating was then conducted on these sample images. This cross-set standardization procedure established a common scale of dissimilarity (DI) between the image pairs of each object set. Finally, we conducted a forced choice recognition memory task (with corresponding targets and lures) to validate our measures of dissimilarity. We used the image pairs that were selected to fully represent the spectrum of dissimilarities, and observed a linear increase in memory performance with increasing dissimilarity between the memory target and the lure. In addition, faster response times were observed on more dissimilar target–lure trials. These observations confirm that our DI is meaningful and predictive of memory performance.

The spatial-arrangement method has been established as a fast and effective method for collecting similarity information (Hout et al., 2016; Hout et al., 2013). However, it has previously been criticized for limiting the number of dimensions on which individuals can represent similarity (Verheyen, Voorspoels, Vanpaemel, & Storms, 2016). First, it is doubtful whether any of the current methods (e.g., spatial arrangement or exhaustive pairwise comparison) completely avoid this limitation (Hout & Goldinger, 2016). Maintaining a consistent and reliable high-dimension approach to rating the similarity of stimuli using an exhaustive pairwise rating system is highly challenging. A lack of perspective for both the breadth of similarity across a set and the nuance relationships within the set would potentially produce ratings equally as noisy as those from the spatial-arrangement method, if not more noisy. Participant fatigue may also impact the accuracy of pairwise ratings, due to the far longer experimentation time required to complete pairwise rating than with the spatial-arrangement method. These factors suggest that the spatial-arrangement method is just as capable as the pairwise comparison method, if not more so, of representing these high dimensions. This is supported by the strong linear correlations between the similarity indices produced by a spatial-arrangement method and by the pairwise rating procedure (Migo et al., 2013). Furthermore, Hout et al. (2013) demonstrated the same equivalence by showing that higher-dimensional similarities could easily arise from the averaging of similarity arrangements across participants who each focused on two different dimensions. Moreover, Hout and Goldinger (2016) calculated that upon consideration of the data collection time (and its associated factors, such as participant fatigue and disengagement), the spatial-arrangement method was more capable of explaining variance in response times on two same–different classification tasks. Taken together, these comparisons illustrate that despite using computer displays restricted to two dimensions, the spatial-arrangement method can effectively represent similarity spanning multiple dimensions across a set of participants.

Despite the efficacy of the spatial-arrangement method in representing multiple dimensions, we further minimized uncertainty regarding potentially unrepresented dimensions by exclusively using grayscale images in SOLID. This essentially eliminated one of the dimensions contributing to variations in sorting. In addition, the images in SOLID are generally more similar than those in previous iterations of similar object image databases (Hout et al., 2014), and provide a finer scale on which dissimilarity is measured. These differences reduce the number of potential factors on which participants could arrange the images, thus reducing individual variability in the dimensions prioritized during sorting. Lastly, the scaling of dissimilarity and behavioral performance on the memory task in the present study illustrates the clear success of our spatial-arrangement procedure and subsequent standardization procedures in representing meaningful image similarity.

Our database offers a wide range of everyday objects, which vary in different ways. Multiple features distinguish the images within each set. For example, some objects are perceptually distinct (e.g., in terms of shape, feature, or orientation), while others can be differentiated by a semantic label (e.g., for types of car: manufacturer, model year, function). This ensures that no single feature can distinguish between a given target and the other possible foils. SOLID affords researchers the ability to choose images (and image groups) on the basis of a single feature or a combination of features, to investigate perceptual versus semantic processing. Researchers who are interested in characterizing memory processes will find this particularly useful, in that it captures nonsystematic variation of the representations to be stored and retrieved from memory. Consequently, these differences in multiple image features preclude participants from using a specific strategy to guide their memory decisions in distinguishing between old and new representations.

The degree of similarity between items is also crucial for studies investigating pattern separation and completion (Bakker et al., 2008; Hunsaker & Kesner, 2013; Lacy et al., 2011; Liu et al., 2016; Norman, 2012). Unlike existing databases (Hout et al., 2014; Migo et al., 2013; Stark, Yassa, Lacy, & Stark, 2013), SOLID allows researchers to systematically create a parametric gradient of similarity between different items (pairs, triplets, or quartets, up to even ten items). Furthermore, systematic manipulation of item similarity could be used to assess item generalization, the similarity threshold at which an old item is judged as new, and whether that threshold can be manipulated experimentally (Kahnt & Tobler, 2016; Motley & Kirwan, 2012). Finally, item similarity could also be used to probe memory recollection and familiarity using different testing formats (Migo et al., 2009; Migo et al., 2014).

The ecological relevance of images of everyday objects is another advantage of using SOLID. Our database enables the investigation of multiple aspects of cognition with control over both perceptual and semantic image features. Furthermore, cognitive abilities, such as episodic memory, can be assessed independently of language. This advantage is especially beneficial for research examining clinical or developmental populations with diminished or incomplete lexical abilities. Deficits in language processing networks attenuate the ability of current clinical assessments (such as the Logical Memory subtest of the Wechsler Memory Scale; Wechsler, 1987) to accurately and reliably determine memory impairment. In spite of their limitations, these assessments are used as inclusion and primary efficacy measures for clinical trials of conditions such as Alzheimer’s disease (Chapman et al., 2016). Similarly, clinical assessments not reliant on language, such as the Doors and People task (Morris, Abrahams, Baddeley, & Polkey, 1995), provide neither the same breadth of realistic images nor the ability to carefully titrate task difficulty according to memory function. The use of SOLID to carry out clinically relevant memory assessments could enable better early identification, classification, and targeting of treatments in clinical populations.

The comparability of image pairs across object sets is a key benefit of SOLID over existing databases. Critically, this will allow for control of image pair similarity in future experiments in which researchers wish to use stimuli from different image sets (e.g., using equally similar pairs of apples and keys). To provide researchers with easy access to different object pairs (or triplets) of equal similarity, we have created two Matlab (The MathWorks Inc., Natick, MA) functions that are available here: https://github.com/frdarya/SOLID. Previous attempts to develop a visual object stimulus database with similarity information have either not provided a common scale of similarity across object sets (Hout et al., 2014) or not provided enough stimuli to support paradigms requiring large numbers of trials (Migo et al., 2013). Enabling this degree of control over similarity is critical for high-level studies of memory and cognition, and combining this strength with the option to use it across many trials will allow research questions to be addressed using neuroimaging techniques. In effectively providing both of these characteristics, SOLID represents a very valuable tool that will allow researchers to better investigate memory, cognition, and their neural bases.

### Author note

We thank Lewis Fry and Harry Hoyle for their contributions to the data collection. We also thank Ellen Migo and Tim Brady for providing access to some of the images available in this database. This work would not have been possible without the support of doctoral training funding awarded to O.G. by the EPSRC, and of a PDS award from the University of Manchester received by D.F. Author contributions: In this study, D.F., O.G., and D.M. designed the experiments; D.F. and O.G. collected and analyzed the data; and D.F., O.G., and D.M. wrote the manuscript. Competing interests: The authors declare no competing interests.

## Electronic supplementary material

ESM 1 (DOCX 13.0 kb)
