# Supplementary material for: SOLID-Similar object and lure image database
Source: Behav Res Methods. 2019 Feb 25;52(1):151–61. doi: 10.3758/s13428-019-01211-7 (PMC7005083; doi:10.3758/s13428-019-01211-7)
Supplement: Supplementary file 1 — (DOCX 13.0 kb) [file 13428_2019_1211_MOESM1_ESM.docx]

Supplementary Materials

The images and dissimilarity matrices are available to download here: <https://www.click2go.umip.com/i/copyright/solid.html>

To download, please add SOLID to your basket, create an account to check out. After agreeing to the terms and conditions an email will be sent with a download link for the database.
